# Socioeconomic inequalities in unmet dental care needs in Spain

**DOI:** 10.3389/froh.2026.1768458

**Published:** 2026-04-10

**Authors:** Sergio Zuniga-Jara, Martin Zuniga-Soria, Sofia Ruiz-Campo, Karla Soria-Barreto

**Affiliations:** 1 School of Business Sciences, Universidad Catolica del Norte, Coquimbo, Chile; 2 Faculty of Medicine, Universidad Catolica del Norte, Coquimbo, Chile; 3 Business Administration Department, Universidad Francisco de Vitoria, Madrid, Spain

**Keywords:** concentration index, oral health disparities, Slope Index of Inequality, socioeconomic inequalities, Spain living conditions survey, unmet dental care needs

## Abstract

**Objective:**

Socioeconomic inequalities in access to dental care remain a critical public health challenge in Spain, where adult coverage is limited and largely financed through out-of-pocket payments. This study aims to estimate the magnitude of socioeconomic inequality in unmet dental care needs among the Spanish adult population in 2024 and to decompose the contribution of its determinants.

**Methods:**

A cross-sectional analysis was conducted using data from the 2024 Spanish Living Conditions Survey (ECV). The analytic sample included 32,425 adults who reported a perceived need for dental care in the previous 12 months. Socioeconomic position was measured using equivalised disposable household income. Income-related inequalities were quantified using the Concentration Index (CI) for relative inequality and the Slope Index of Inequality (SII) for absolute inequality. Furthermore, a Wagstaff decomposition analysis was performed to isolate the specific contribution of multidimensional factors such as material deprivation and housing tenure.

**Results:**

Among individuals with perceived need, 10.44% reported unmet dental care needs, with economic constraints identified as the overwhelming barrier (78.9%). The analysis revealed a robust pro-poor gradient, indicated by a Concentration Index of −0.361 (*p* < 0.001). The Slope Index of Inequality was −0.237 (*p* < 0.001), reflecting an absolute gap of approximately 23.7 percentage points in the probability of unmet needs between the lowest and highest income extremes. Decomposition analysis identified material deprivation as the primary driver, explaining 28.0% of the total inequality, followed by social class (11.1%) and education (7.2%).

**Conclusion:**

The findings confirm that the “Inverse Care Law” persists in the Spanish dental healthcare system, driven fundamentally by structural economic vulnerability rather than demographic factors. The current model fails to protect the most deprived groups. Policy interventions must prioritize the expansion of public coverage and implement targeted financial protection mechanisms to effectively reduce these inequities.

## 1 Introduction

Oral health is an integral part of general health and a fundamental human right, influencing not only physical well-being but also mental health (1). Yet, it remains one of the most neglected areas of global health policy (2). Despite significant improvements in oral health indicators over the last decades (3), socioeconomic inequalities in access to dental care persist as a widespread challenge across high-income countries (4). In Europe, dental care is frequently excluded from the statutory coverage of national health systems or subject to high co-payments, transforming it into a “luxury good” whose consumption is highly sensitive to household income (5, 6). This structural exclusion generates a “pro-rich” gradient in access, where vulnerable populations (who often bear the highest burden of oral disease) face the greatest barriers to care, a phenomenon famously described by (7) as the “Inverse Care Law.”

The case of Spain is particularly paradigmatic within this context. Although Spain boasts a universal National Health System (SNS) that provides comprehensive coverage for medical care, adult dental services are largely excluded from the public benefits basket, covering only acute emergencies and diagnostic procedures (3, 8). Consequently, the vast majority of dental care is provided by the private sector and financed through out-of-pocket payments. This financing model places the full burden of cost on households, frequently leading to catastrophic health expenditures (9), and creating a fertile ground for inequality. This financing model places the full burden of cost on households, creating a fertile ground for inequality. Previous studies using data from the European Union Statistics on Income and Living Conditions (EU-SILC) have consistently identified Spain as having one of the highest levels of income-related inequality in unmet dental needs among European nations (4, 10).

However, the existing literature on this topic in Spain presents certain limitations. Most prior research relies on data predating the post-pandemic context or focuses on general inequality indices without deeply exploring the structural determinants driving these disparities. For instance, the longitudinal analysis by Urbanos-Garrido (10) examined trends up to 2017, noting that inequalities exacerbated during economic downturns. Yet, little is known about the persistence of these barriers in the current landscape, specifically regarding how multidimensional factors (beyond income) such as material deprivation and housing tenure intersect to hinder access. Furthermore, previous studies often analyzed the general population, which may dilute the measurement of access barriers by including individuals who simply do not need care (11).

### 1.1 Research problem and objective

This study addresses these gaps by analyzing the most recent data available for Spain. The central research problem is to quantify the extent to which the current mixed-market model fails to protect the most vulnerable adults from unmet dental needs. The primary objective of this paper is to estimate the magnitude of socioeconomic inequality in unmet dental care needs among the Spanish adult population in 2024 and to decompose the contribution of its determinants.

### 1.2 Hypothesis

We hypothesize that, despite economic recovery periods, access to dental care in Spain remains structurally unequal, driven primarily by financial constraints and material deprivation rather than by demographic factors or differences in need. Specifically, we posit that the “Inverse Care Law” persists, with a robust pro-poor concentration of unmet needs that cannot be explained solely by income but is deeply rooted in broader conditions of economic vulnerability.

### 1.3 Methodological approach and contribution

To test this hypothesis, we utilize data from the 2024 Spanish Living Conditions Survey (ECV). Our methodological approach advances beyond descriptive statistics by employing rigorous inequality metrics: the Concentration Index (CI) to measure relative inequality and the Slope Index of Inequality (SII) to quantify the absolute gap. Crucially, we apply a Wagstaff decomposition analysis to isolate the specific weight of determinants such as material deprivation, social class, and housing tenure.

This study contributes to the literature in three key ways. First, it provides an updated post-pandemic snapshot of dental health equity in Spain. Second, by restricting the analysis to the population with “perceived need,” we offer a more precise measure of access barriers, avoiding the selection bias present in general population studies. Third, the decomposition analysis moves from merely describing that inequality exists to explaining why it exists, offering concrete evidence to inform policy debates on the expansion of public dental coverage in Spain.

## 2 Materials and methods

### 2.1 Design and data source

This cross-sectional study utilizes data from the 2024 Spanish Living Conditions Survey (Encuesta de Condiciones de Vida, ECV), conducted by the National Statistics Institute (INE). The ECV is the Spanish component of the European Union Statistics on Income and Living Conditions (EU-SILC). The survey employs a probabilistic, stratified, multistage sampling design based on census registers, ensuring representativeness at both the national level and the level of Autonomous Communities. The 2024 wave included approximately 13,000 households. Data collection was carried out between April and September 2024 via face-to-face and telephone interviews.

### 2.2 Study population

**Figure 1:**
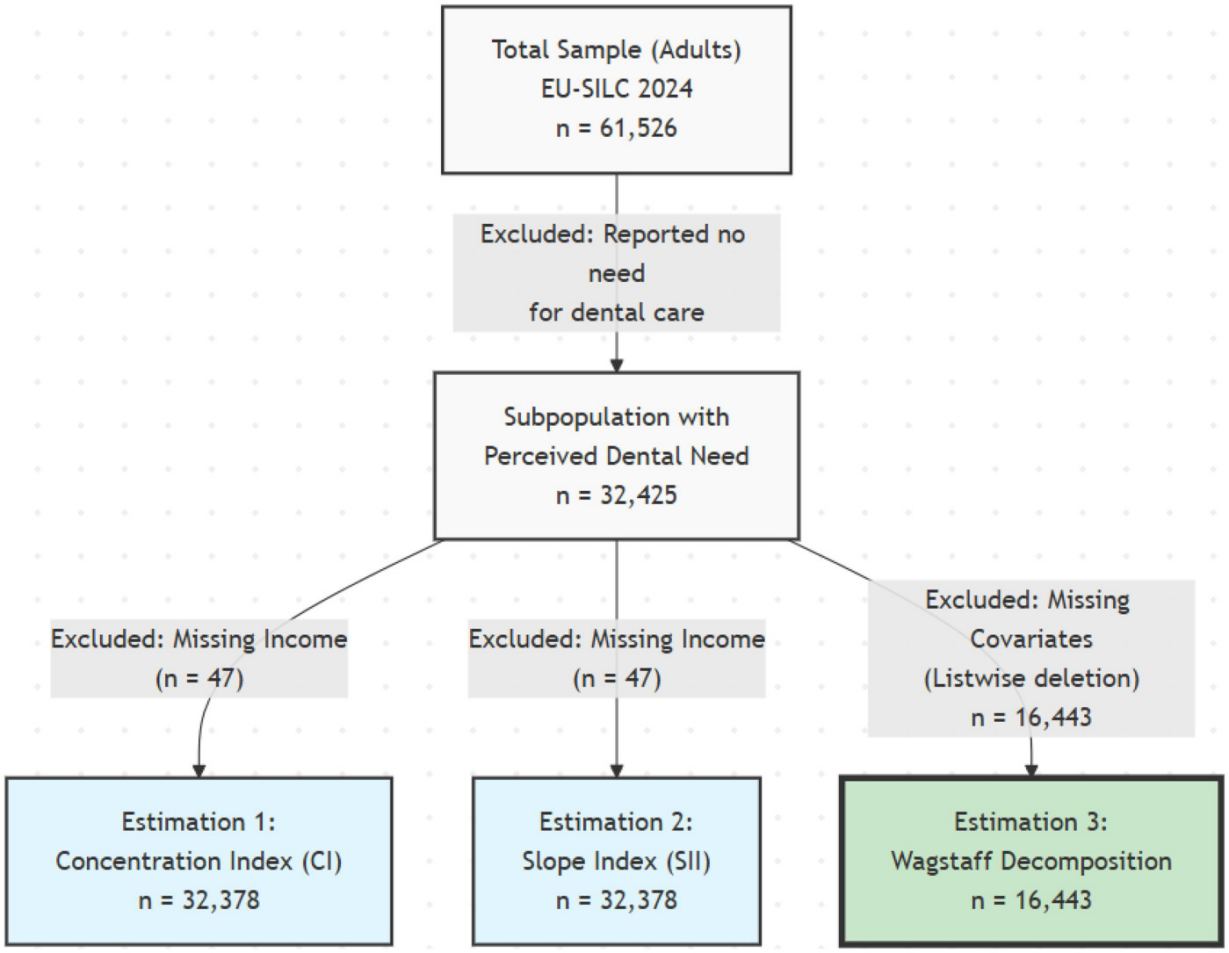
Flowchart of the study population selection and derivation of the final analytic sample (ECV Spain, 2024).

The analytic sample was derived through a sequential filtering process (see Figure 1). From the initial sample of 61,526 adults, the study focused exclusively on individuals who reported a perceived need for dental care in the previous 12 months (Question PH060_F; *n* = 32,425). Individuals who reported no need for dental care were excluded to ensure that the analysis of access barriers was performed on the relevant population at risk. The final analytic sample for the multivariate decomposition analysis was restricted to individuals with complete data on all covariates (*n* = 16,443), following a complete case analysis approach.

### 2.3 Variables

#### 2.3.1 Outcome Variable

The primary outcome was unmet dental need, defined as a binary variable based on Question PH060. Participants who reported needing dental examination or treatment but did not receive it “every time it was needed” were classified as having an unmet need (1 = Unmet Need; 0 = Met Need). Among those reporting unmet needs, the main reason for non-access was categorized based on Question PH070, distinguishing between economic barriers (“Could not afford it”) and non-monetary reasons (e.g., fear, waiting lists, or lack of time).

#### 2.3.2 Socioeconomic stratification

Socioeconomic position was determined using equivalised disposable household income. Total household income (Question HY020) was divided by the number of consumption units in the household (Question HX040), applying the OECD-modified equivalence scale to adjust for household size and composition. Negative income values were excluded. Based on this continuous variable, individuals were ranked from poorest to richest to generate: (1) income quintiles for descriptive stratification, and (2) fractional ranks (ridit scores) for inequality indices.

#### 2.3.3 Covariates

To examine the determinants of inequality, the following sociodemographic and health variables were included: sex, age group (16–34, 35–64, 65+), education level (ISCED classification), self-rated general health (good/fair vs. poor/very poor), housing tenure (owner vs. tenant), severe material deprivation (structural poverty indicator), and occupational social class (derived from ISCO-08 codes).

### 2.4 Statistical analysis

All analyses were performed using Stata v.15 (StataCorp LLC, College Station, TX, USA). We applied calibrated sampling weights (PB040) to all descriptive statistics and regression models to ensure population representativeness.

#### 2.4.1 Measurement of inequality

To quantify health inequality, we employed two complementary indices:

− Concentration Index (CI): Used to measure relative inequality. The CI quantifies the extent to which unmet dental needs are concentrated among the socioeconomically disadvantaged. It was estimated using the “convenient regression” approach proposed by Kakwani et al. (12). A negative CI indicates that unmet needs are disproportionately concentrated among the poor.
− Slope Index of Inequality (SII): Used to measure absolute inequality. The SII represents the absolute difference in the predicted probability of unmet need between the hypothetical poorest (R = 0) and richest (R = 1) individuals. It was estimated using a generalized linear model (identity link) with robust standard errors to correct for heteroscedasticity, regressing the binary outcome on the fractional rank (ridit score) of income (13).

#### 2.4.2 Decomposition analysis

To identify the specific factors driving income-related inequalities, we performed a decomposition of the Concentration Index following the methodology by Wagstaff et al. (14) and O'Donnell et al. (15). This method relies on a linear regression model where the outcome $y_{i}$ is the binary variable of unmet need, and $x_{ki}$ represents a set of k social and health determinants is expressed as:

$$y_{i}\;=\;\alpha \;+\;s\overset{}{\underset{k}{\sum }}\beta_{k}\;x_{ki}\;+\;\varepsilon_{i}$$

The overall Concentration Index (C) is then decomposed into the sum of the contributions of each determinant k was decomposed as:

$$C\;=\;\sum_{k}\left(\frac{\beta_{k}{\bar{x}}_{k}}{\mu }\right)C_{k}+\frac{GC_{e}}{\mu }$$

where:

$\beta_{k}$ is the regression coefficient of determinant k (its impact on unmet need).

$\underset{k}{x}$ is the mean of determinant *k*.

μ is the mean of the outcome variable (unmet need).

$C_{k}$ is the Concentration Index of determinant k (how unequally that factor, e.g., material deprivation, is distributed between the rich and the poor).

$\frac{GC_{e}}{\mu }$ is the residual component (the unexplained part).

This analysis allows for the calculation of the percentage contribution of each factor (e.g., income, material deprivation, housing) to the total observed inequality in dental access.

## 3 Results

### 3.1 Study population and sample selection

The sample selection process is illustrated in Figure 1. From the initial sample of 61,526 adults in the 2024 ECV, 32,425 individuals (52.7%) reported a perceived need for dental care during the previous 12 months. This subgroup constituted the target population for the analysis of access barriers. For the multivariate decomposition analysis, the sample was further restricted to 16,443 individuals due to listwise deletion of cases with missing data on covariates (primarily income and material deprivation).

### 3.2 Sociodemographic characteristics and barriers to access

Table 1 summarizes the prevalence of dental needs and the reasons for non-access. Among those who required dental care, 10.44% (*n* = 3,386) reported having unmet needs (i.e., they did not receive care every time it was needed).

**Table 1:** Prevalence of perceived dental needs, effective access, and self-reported reasons for non-access in Spain (weighted estimates).

Indicator	Frequency (*n*)	Percentage (%)
Perceived dental need (Last 12 months)	61,526	100.00
No need	28,565	46.43
Did not answer/unknown	536	0.87
Yes (need)	32,425	52.70
Access to care (among those with need)	32,425	100.00
Need met (received care)	29,039	89.56
Need unmet (did not receive care)	3,386	10.44
Main reason for unmet need	3,377^a^	100.00
Economic (Too expensive)	2,664	78.89
Fear of dentist	160	4.74
No time available	106	3.14
Waiting list	68	2.01
Chose to wait/spontaneous improvement	70	2.07
Too far to travel	11	0.33
Did not know a good dentist	12	0.36
Other reasons	286	8.47

Data source: Spanish Living Conditions Survey (ECV) 2024. *n* represents the unweighted sample size; percentages (%) are calculated using calibrated sampling weights to reflect the Spanish adult population.

^a^ Missing values for the specific reason (*n* = 9) were excluded from the percentage calculation.

When identifying the main barrier to access, economic constraints were the overwhelming determinant. Financial reasons (“Too expensive”) accounted for 78.89% of all unmet needs, whereas non-monetary barriers such as fear of the dentist (4.74%), lack of time (3.14%), or waiting lists (2.01%) played a marginal role.

Table 2 compares the sociodemographic profiles of individuals with met vs. unmet dental needs. The bivariate analysis reveals a marked socioeconomic gradient:

- Income: Individuals with unmet needs had a significantly lower mean equivalised income (€16,555) compared to those who received care (€25,137; *p* < 0.001).
- Material Deprivation: The prevalence of severe material deprivation was seven times higher in the unmet need group (27.9%) compared to the met need group (3.9%; *p* < 0.001).
- Social Class and Education: Unmet needs were significantly more frequent among the working class and those with lower educational attainment.
- Housing: Renters reported unmet needs more frequently (28.0%) than homeowners (12.2%).
- Health Status: Individuals with poor or very poor self-rated health were significantly more likely to report unmet dental needs (18.3% vs. 6.4%).
- Notably, no statistically significant difference was observed by sex (*p* = 0.365), indicating that men and women face similar levels of unmet need when other factors are not controlled.

**Table 2:** Sociodemographic characteristics of the study population stratified by met and unmet dental care needs (weighted analysis).

Characteristic	Met need (*n* = 29,001)	%	Unmet need (*n* = 3,380)	%	*P*-value^a^
Sex					0.365
Men	13,096	45.2	1,554	46	
Women	15,905	54.8	1,826	54	
Age group					<0.001
16–34	5,757	19.9	460	13.6	
35–64	15,762	54.3	2,126	62.9	
65+	7,482	25.8	794	23.5	
Education level					<0.001
Low (primary or less)	3,858	13.3	811	24	
Medium (secondary)	13,797	47.6	1,858	55	
High (tertiary)	11,330	39.1	710	21	
Social class					<0.001
High/professional	4,364	29.1	177	12.2	
Middle	6,756	45	664	45.8	
Working	3,877	25.9	610	42	
Income quintile					<0.001
Q1 (poorest)	3,839	13.2	1,204	35.6	
Q2	4,864	16.8	848	25.1	
Q3	5,846	20.2	610	18	
Q4	6,592	22.7	482	14.3	
Q5 (richest)	7,857	27.1	236	7	
Material deprivation					<0.001
Severe deprivation (yes)	1,143	3.9	944	27.9	
No Deprivation	27,858	96.1	2,436	72.1	
Housing tenure					<0.001
Renting	3,524	12.2	948	28	
Owned/other	25,477	87.8	2,432	72	
Self-rated health					<0.001
Poor/very poor	1,842	6.4	620	18.3	
Good/fair	27,159	93.6	2,760	81.7	
Equivalised Income (€)	25,136.80	(Mean)	16,555.10	(Mean)	<0.001

*n* represents the unweighted sample size; percentages (%) are calculated using calibrated sampling weights to reflect the Spanish adult population.

^a^ *P*-values calculated using Chi-square test for categorical variables and *t*-test for continuous variables.

### 3.3 Magnitude of socioeconomic inequality

The analysis of health inequality indices confirms that unmet dental care needs are disproportionately concentrated among the most disadvantaged population.

**Figure 2:**
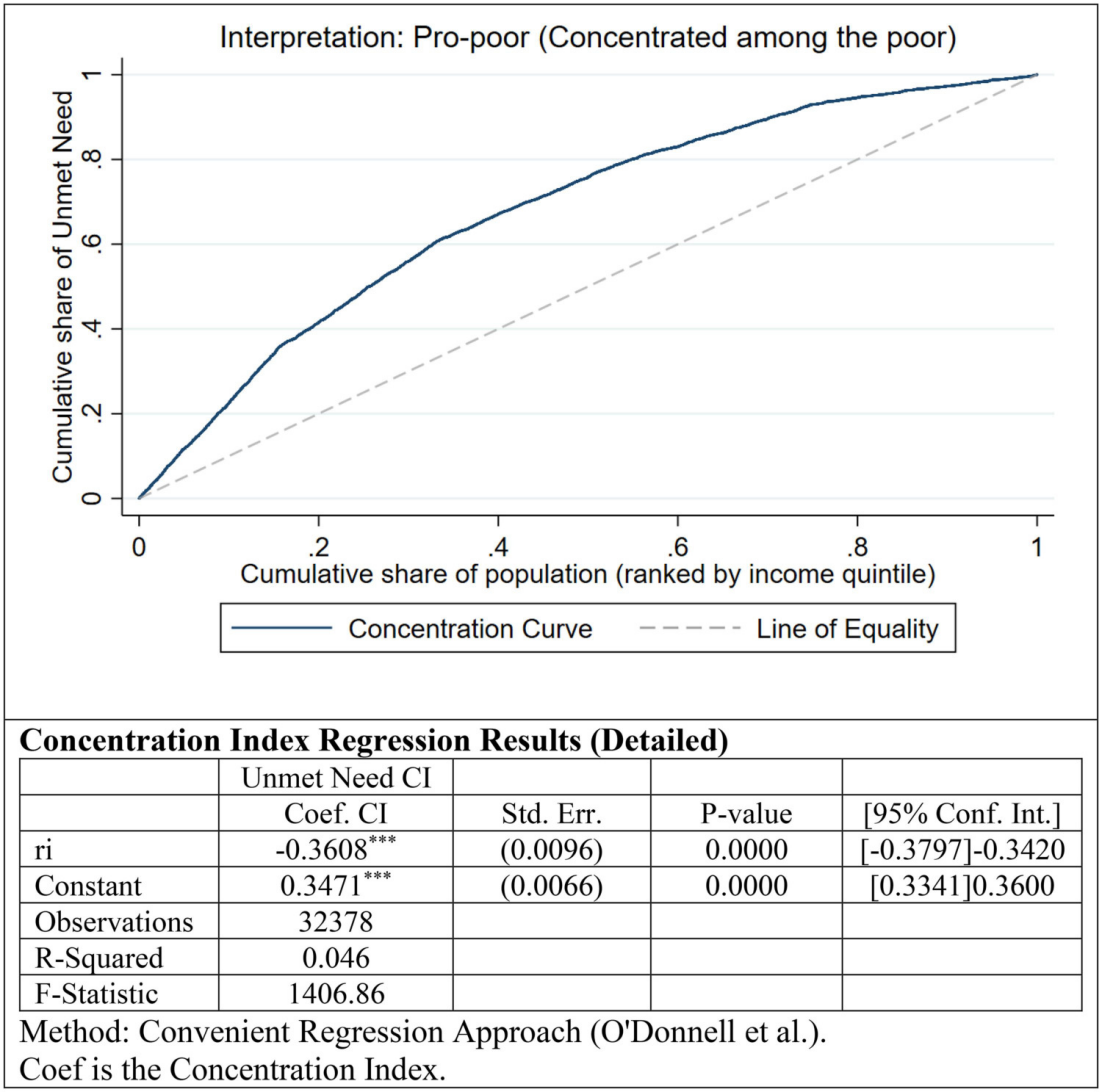
Concentration curve of unmet dental care needs: graphic representation of relative inequality (weighted population).

**Figure 3:**
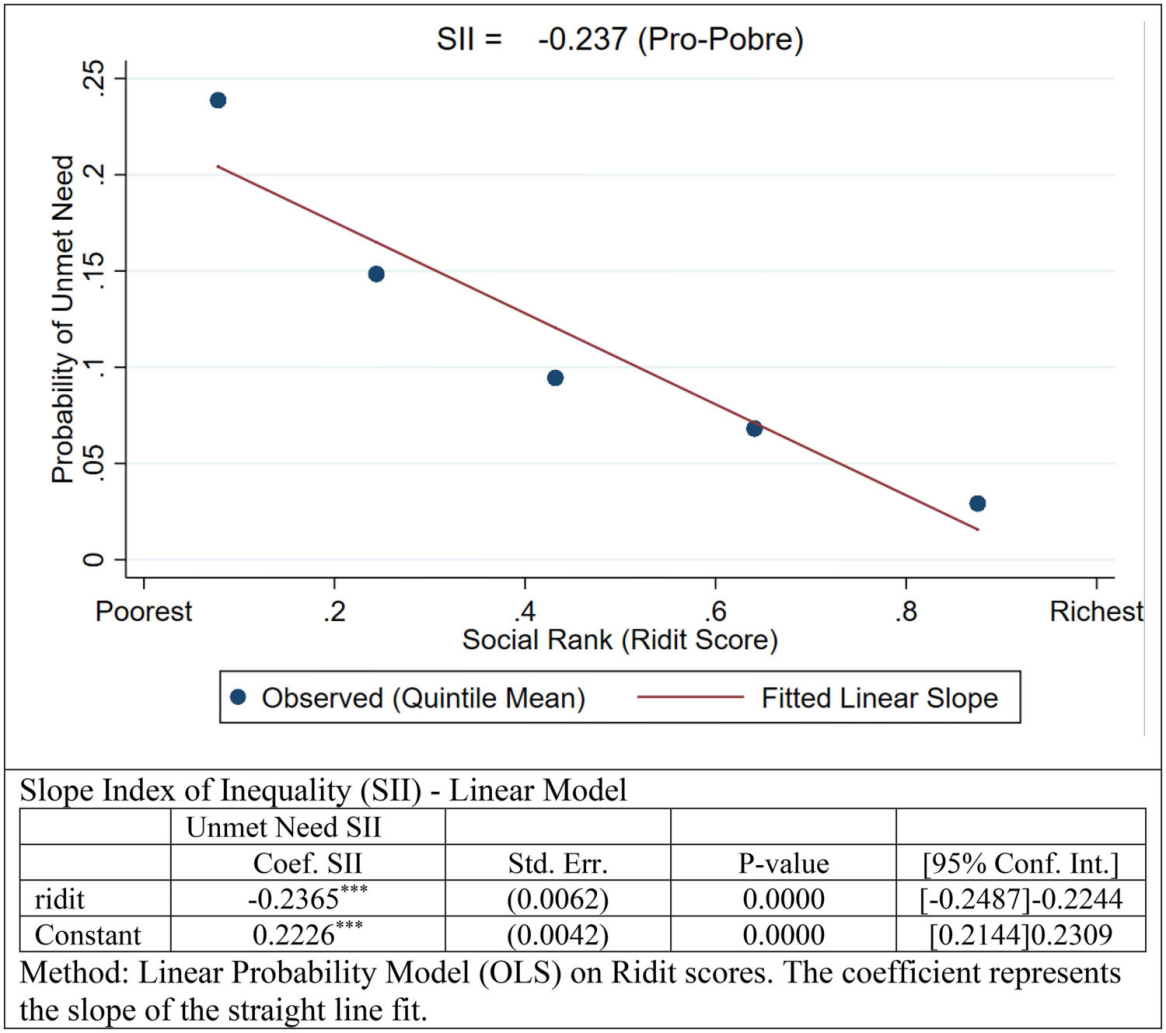
Slope Index of inequality (SII): absolute gap in the probability of unmet dental needs across the income hierarchy (weighted population).

- Relative Inequality (Concentration Index): The Concentration Curve (Figure 2) lies entirely above the line of equality, visually demonstrating that the cumulative share of unmet needs is higher among the poorer segments of the population. The estimated Concentration Index (CI) was −0.361 (*p* < 0.001). The negative sign and the magnitude of the coefficient indicate a high degree of pro-poor inequality, confirming that economic disadvantage is a strong predictor of lack of access.
- Absolute Inequality (Slope Index): The Slope Index of Inequality (SII), estimated via a linear probability model on fractional ranks (Figure 3), yielded a coefficient of −0.237 (*p* < 0.001). This value indicates a substantial absolute gap: the probability of experiencing unmet dental needs is approximately 23.7 percentage points higher for an individual at the bottom of the socioeconomic hierarchy compared to one at the top. The linear fit in Figure 3 illustrates a steep downward gradient, where the risk of unmet need decreases consistently as social rank increases.

### 3.4 Decomposition of inequality

**Figure 4:**
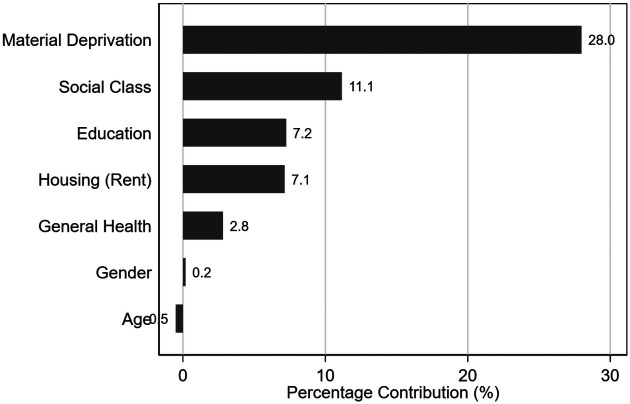
Wagstaff decomposition of the concentration Index: relative contribution of socioeconomic and demographic determinants to inequality (weighted analysis).

To understand the factors driving the observed inequality, a Wagstaff decomposition of the Concentration Index was performed. Figure 4 presents the relative contribution of each determinant to the total inequality.

The results show that the inequality is predominantly structural and economic rather than demographic:

- Material Deprivation was the largest single contributor, explaining 28.0% of the total inequality.
- Social Class and Education contributed 11.1% and 7.2%, respectively.
- Housing Tenure (renting) accounted for 7.1% of the inequality.
- Income (residual contribution after controlling for other variables) explained the largest portion of the remaining variance.

In contrast, demographic factors such as Gender (0.2%) and Age (−0.5%) had negligible or null contributions. This suggests that the “pro-poor” concentration of unmet dental needs in Spain is almost entirely driven by the unequal distribution of economic resources and living conditions, rather than by biological or age-related differences in the need for care.

## 4 Discussion

This study provides robust evidence that the “Inverse Care Law” (7) remains a defining characteristic of the Spanish dental healthcare system. Our findings reveal a profound socioeconomic gradient in access, where the burden of unmet needs falls disproportionately on the most vulnerable. The estimated Concentration Index (−0.361) and Slope Index of Inequality (−0.237) corroborate that this inequality is not random but structurally determined. Specifically, the absolute gap of nearly 24 percentage points between the social extremes highlights a systemic failure to ensure equitable access to oral health, a fundamental component of general well-being.

- Interpretation of Determinants and Mechanisms. A key contribution of this study is the decomposition of inequality, which offers a nuanced understanding of the drivers of exclusion. While previous literature often highlights the role of oral health literacy (education) in access, our Wagstaff decomposition reveals that Material Deprivation (28.0%) is the single largest contributor to inequality, far outweighing Education (7.2%). This suggests that in the current Spanish context, the primary barrier is not a lack of knowledge or “culture” regarding oral health, but rather a direct lack of financial capacity.

This finding aligns with the observation that nearly 80% of unmet needs are attributed to cost. In Spain, adult dental care is largely excluded from the statutory National Health System (SNS) coverage, forcing the population to rely on the private sector. Consequently, dental care behaves as a “luxury good” with high price elasticity; for households experiencing material deprivation, oral health expenses are likely the first to be cut when budgets are tight. This creates a “poverty trap” where economic vulnerability leads to dental deterioration, which in turn may reinforce social exclusion.

### 4.1 Comparison with previous evidence

Our results update and reinforce the trends observed by Urbanos-Garrido (10) and Calzón Fernández et al. (16) regarding the pro-rich bias in unmet dental needs in Spain. While Calzón Fernández et al. documented how the economic crisis exacerbated these gaps, our 2024 data confirm that these inequalities persist even after the economic recovery periods, suggesting they are structural rather than merely cyclical. Furthermore, our findings are consistent with comparative European studies (5, 17–19) which consistently identify Spain as having one of the highest levels of inequality in dental access in the EU, largely due to the limited scope of public coverage compared to countries with broader insurance schemes like Germany or France.

### 4.2 Limitations

These findings must be interpreted in light of certain limitations. First, the cross-sectional design prevents establishing causal relationships between socioeconomic status and unmet needs. Second, the variable of interest relies on “self-perceived need.” It is possible that individuals in the most disadvantaged groups underestimate their need for care due to a normalization of poor oral health (“adaptive preference”), which would imply that our estimates of inequality are actually conservative (underestimated). Finally, the lack of clinical variables (e.g., DMFT index) limits our ability to correlate access barriers with specific clinical outcomes.

## 5 Conclusions and policy implications

The socioeconomic inequality in unmet dental care needs in Spain is high, significant, and fundamentally driven by economic constraints. The dominance of material deprivation and cost as the primary drivers of exclusion indicates that the current model (based on private provision and out-of-pocket payments) provides insufficient financial protection for the adult population.

### 5.1 Policy implications

Policy interventions must move beyond behavioral education or promotion campaigns, which our analysis suggests would have a limited impact on reducing inequality. Instead, effective solutions require structural changes to the financing model:

1. Expansion of Public Coverage: There is an urgent need to progressively expand the Cartera de Servicios (public benefits basket) for adults, prioritizing restorative and preventive treatments that are currently unaffordable for low-income groups.
2. Targeted Financial Protection: Given the strong concentration of unmet needs among the materially deprived, a targeted voucher system or fully subsidized care for low-income households could be a cost-effective short-term mechanism to reduce the absolute gap (SII).
3. Regulation: Policy debates should also consider the regulation of private dental prices or the introduction of sliding-scale fees to mitigate the regressive nature of out-of-pocket payments.

In summary, ensuring the right to health in Spain requires dismantling the financial walls that currently segregate dental care, transforming it from a market privilege into an accessible public good.

## Data Availability

Publicly available datasets were analyzed in this study. This data can be found here: https://www.ine.es/dyngs/Prensa/ECV2024.htm.
